# Optimization of production and quality evaluation of maize‐based snack supplemented with soybean and tiger‐nut (*Cyperus esculenta)* flour

**DOI:** 10.1002/fsn3.359

**Published:** 2016-04-06

**Authors:** Olugbenga O. Awolu, Olufunmilayo S. Omoba, Olumide Olawoye, Modupe Dairo

**Affiliations:** ^1^ Department of Food Science and TechnologyFederal University of TechnologyAkureNigeria; ^2^ Quintas Renewable Energy Solutions LtdAkureNigeria

**Keywords:** Amino acid profile, optimization, pasting, proximate, snack toasted maize

## Abstract

Optimization of the production and evaluation of the quality of maize‐based snack supplemented with soy and tigernut flour was carried out. Experimental design for the composite flour was carried out using optimal design model of response surface methodology. The variables were toasted maize flour (75–85%), soy flour (10–20%), and tigernut flour (5–10%); while the responses were proximate composition and mineral contents. Three blends were selected from optimization results; runs 2, 7, and 11. The functional properties, pasting properties, antioxidant, antinutritional, and amino acid profile of the three blends were evaluated. In addition, sensory evaluation of the cookies produced from the three blends was determined. The results (75/100 g toasted maize flour, 20/100 g soy flour, and 5/100 g tigernut flour) had 16.4/100 g protein, 4.2/100 g ash, 3.5/100 g moisture, 58.5/100 g carbohydrate, 3.0/100 g crude fiber, 14.4/100 g fat, 30.20 ppm calcium, 38.90 ppm potassium, 0.25 ppm manganese, 1.91 ppm iron, 0.14 ppm copper, and 0.98 ppm zinc contents. It also had best overall acceptability.

## Introduction

Cookies are widely consumed all over the world and they represent the largest category of snack foods in most parts of the world. Generally, they are high in carbohydrates, fat, and calorie, but low in fiber, vitamin, and mineral. Currently, fortification of snacks has evolved to enhancing their nutritional quality (Neha and Ramesh 2012; Awolu et al. 2015a,b).

Maize is the most important cereal in the world after wheat and rice with regard to cultivation areas and total production (Osagie and Eka 1998). It has been discovered that maize is nutritionally superior to other cereals in many ways, except in protein value. Maize is low in two essential amino acids, lysine and tryptophan. The addition of soybeans which are rich in lysine and tryptophan was meant to enhance the nutritional capacity of the composite flour.

Soybean is an important legume. It has been found that soybean is a very rich source of protein and fat, phytochemicals, and minerals such as copper, zinc, and manganese (Nwokolo 1996). Tigernut is an underutilized crop reputed to be very rich in minerals such as calcium, magnesium and iron (Omode et al. 1995; Oladele and Aina 2007). Tigernuts have good dietary fiber, carbohydrate contents, and it is a rich source of antioxidant (Umerie and Enebeli 1997). Currently, the use and acceptability of tigernut in food formulation is on the increase (Ade‐Omowaye et al. 2008; Omoba et al. 2015).

This study aimed at utilizing these rich crops in order to develop composite flour that is maize (toasted) based. Toasting had been found to enhance energy value, antioxidant capacity and calcium, sodium, magnesium, and zinc contents of maize (Oboh et al. 2010). The effect of the composite flour composition on the dependent variables was evaluated. The functional properties, antioxidant properties, and amino acid profile of the chosen best blends were determined. Cookies were produced and the sensory properties of the cookies were evaluated in order to determine its sensory acceptability.

## Materials and Methodology

### Materials

Maize grains, soy beans, and tigernut tubers were obtained from Akure, Nigeria. All chemicals were of analytical grade.

### Toasted maize (corn) flour production

Dried white‐maize grains were properly sorted, toasted at 150–200°C until a uniform golden brown color was obtained. The toasted grains were allowed to cool and milled into fine powder. The powder was sieved using a 2‐mm mesh sieve in order to obtain the toasted corn flour (A).

### Soy flour production

Soy flour (B) was produced according to the method of IITA, 1990.

### Tigernut flour production

Tigernut flour (C) was produced according to the method of Adejuyitan (2011).

### Experimental design and statistical analyses

Optimal mixture design of response surface methodology (Design Expert 8.0.3.1., Stat‐Ease Inc., Minneapolis, USA trial version) was used. The variables were toasted corn flour (75–85%), soy flour (10–20%), and tigernut flour (5–10%); which generated 16 experimental runs. The responses were proximate and minerals compositions. Three best samples obtained from the optimization result were used for further analyses; functional properties, pasting characteristics, antioxidant and antinutritional evaluations, amino acid profile, cookies production, and sensory evaluation.

### Proximate analysis

The proximate composition of the flour blend samples was determined using AOAC 2005 methods. The samples were analyzed for moisture, ash, crude fiber, crude protein, and crude fat, whereas carbohydrate was calculated by difference.

#### Determination of crude protein

Protein content was determined using Kjeldahl method, according to the procedure of AOAC (2005). Concentrated H_2_SO_4_ (12 mL) and two tablets of selenium catalyst were put into a Kjeldahl digestion flask containing 1 g of the sample. The flask was placed in the digester in a fume cupboard, switched on and digested for 45 min to obtain a clear colorless solution. The digest was distilled with 4% boric acid, and 20% sodium hydroxide solution was automatically metered into it in the distillation equipment until distillation was completed. The distillate was then titrated with 0.1‐mol/L HCl until a violet color was formed, indicating the end point. A blank was run under the same condition as with the sample. Total nitrogen content was then calculated using equation (1).

(1) $$\begin{array}{c} \text{Protein}=\frac{[\text{titre value of sample‐blank}]\times 0.01\times 14.007\times 6.25}{1000\times \text{weight of sample}}\times 100 \end{array}$$

#### Determination of ash content

Two grams of samples were weighed into well incinerated crucibles and then ashed in a muffle furnace at 600 °C for 3 h. The ash content was calculated using equation (2).

(2) $$\text{Ash content}=\frac{\text{weight of ash}}{\text{weight of sample}}\times 100$$

#### Determination of moisture content

Moisture content was determined using AOAC (2005) method. About 5 g of each sample was weighed into petri dishes of a known weight. It was then dried in the oven at 105 ± 1°C for 4 h. The samples were cooled in a desiccator and weighed. The moisture content was calculated applying equation (3)

(3) $$\begin{array}{cc} & \text{Percentage moisture content}\\ & =\frac{\text{change in weight}}{\text{weight of food before drying}}\times 100 \end{array}$$

#### Determination of crude fiber

Crude fiber was determined using the AOAC (2005) method. About 5 g of each sample was weighed into a 500‐mL Erlenmeyer flask and 100 mL of trichloroacetic acid digestion reagent added. It was then brought to boiling and refluxed for exactly 40 min counting from the start of boiling. The flask was removed from the heater, cooled a little, and filtered through a 15.0‐cm Whatman paper. The residue was washed with hot water stirred once with a spatula and transferred to a porcelain dish. The sample was dried overnight at 105 °C. After drying, it was transferred to a desiccator and weighed W_1_ when cool. It was ashed in a muffle furnace at 500°C for 6 h, allowed to cool, and reweighed W_2_. The percentage crude fiber was calculated as shown in equation (4)

(4) $$\%\text{crude fibre}=\frac{W_{1}-W_{2}}{W_{0}}\times 100$$

W_2_ = Weight of crucible + fiber + ash, W_1_ = Weight of crucible + ash, W_0_ = Dry weight of food sample.

#### Determination of fat content

Fat content was determined using AOAC (2005) method. About 2 g of each sample was weighed on a chemical balance and wrapped in a filter paper. It was then placed in an extraction thimble. Extractor was cleaned, dried in an oven, and cooled in the desiccators before weighing. Then, 25 mL of N‐hexane was measured into the round bottom; the fat content was extracted with this solvent. After extraction, the solvent was evaporated by drying in the oven. The flask and its contents were then cooled in a desiccator and weighed fat content. The percentage fat content was calculated as shown in equation (5)

(5) $$\text{Fat content}\left(\%\right)=\frac{\text{weight of fat extracted}}{\text{weight of food sample}}\times 100$$

### Analyses of mineral compositions

The method described by AOAC (2005) was used for minerals analysis. The ash was digested with 3 cm^3^ of 3 mol/L HCl and made up to the mark in a 100 cm^3^ standard flask with 0.36 mol/L HCl before the mineral elements were determined by atomic absorption spectrophotometer (Buck scientific 210 VGP, Bulk Scientific Inc., 06855 USA). The following elements were determined for each of the 16 samples; Calcium, Potassium, Manganese, Iron, Copper, and Zinc.

### Determination of functional properties

The best three samples selected based on the proximate and mineral analyses of all the samples were subjected to functional properties analyses.

#### Bulk density (BD)

Bulk density was estimated as described by Maninder et al. (2007).

#### Swelling index

The method of Ukpabi and Ndimele (1990) was used.

#### Foaming capacity and foam stability

The method described by Narayana and Narasinga Rao (1982) was used.

#### Water and oil absorption capacities

Water absorption capacity of flours blends was measured according to the centrifugation method (AOAC 2005).

#### Least gelation concentration

The least gelation concentration (LGC) was evaluated by using the method of Coffman and Garcia (1977) with some modifications. Appropriate sample suspension of 0.1, 0.2, 0.3, 0.4, 0.5, 0.6, 0.7, and 0.8 g was weighed into 5‐mL distilled water each to make 20% (w/v) suspension. The test tubes containing theses suspensions were heated at 90°C for 1 h in water bath. The contents were cooled under tap water and kept for 1 h at 10°C. The LGC was determined as that concentration when the sample from inverted tube did not slip equation (6)

(6) $$\text{LGC}\%=\frac{\text{weight of sample}}{\text{5ml of water}}1\times 100$$

### Determination of pasting properties

The selected three flour blends were subjected to rapid visco‐analysis. Xanthan gum (0.3 g) was added to each of the samples and properly mixed. The flour sample (3 g) was mixed with 25‐mL distilled water in the canister of a rapid visco‐analyzer (RVA, model 3D; Newport Scientific, Sydney, Australia) monitored with RVA control software and operated.

### Antioxidant and antinutritional properties determination

Oxalate was determined by the method of Day and Underwood (1986), whereas phytate was determined by the method of Major et al. 1990. Total Phenol was determined by the method of AOAC (2005).

### Amino acid profile

Samples were prepared for amino acid determination by acid hydrolysis. Hydrochloric acid (6 N) was used to hydrolyze the sample for 24 h at 110°C in a vial under vacuum and N_2_ atmosphere. The sample solution was evaporated and dissolved in sodium citrate buffer (pH 2.2). The hydrolysates were analyzed by a post‐column derivative method using a HPLC, which was combined with a Pickering PCX5200 derivatizer (Pickering Laboratories, Inc., USA) and ion exchange column (3.0 × 250 mm, 8 *μ*m). The amino acids were spectrophotometrically identified by measuring at 570 nm.

### Cookies production

Twenty grams of hydrogenated fat and 30 g of powdered sugar were creamed together at medium speed until light and fluffy appearance was formed. Then 100 g of the composite flour sample was added with 0.3 g of xanthan gum, 1.7 g of salt, and 1.7‐g baking powder (ammonium bicarbonate) and mixed together until uniform smooth dough was obtained. The dough was allowed to rest for 20 min and then rolled out to 10 mm thickness on a board and cut into round shape about 75 cm diameter with a biscuit cutter. The cookies were placed in a greased baking tray and baked in a preheated oven at 180°C for 10 min.

### Sensory evaluation

Sensory analysis was carried out on the cookies produced from each of the flour blends. Ten members trained panelists were involved. Quality attributes evaluated were color, texture, taste, flavor, appearance, crispiness, and overall acceptability using a 9‐point Hedonic scale, where 9 is like extremely and 1 is dislike extremely.

## Results and Discussion

### Proximate composition

**Figure 1:**
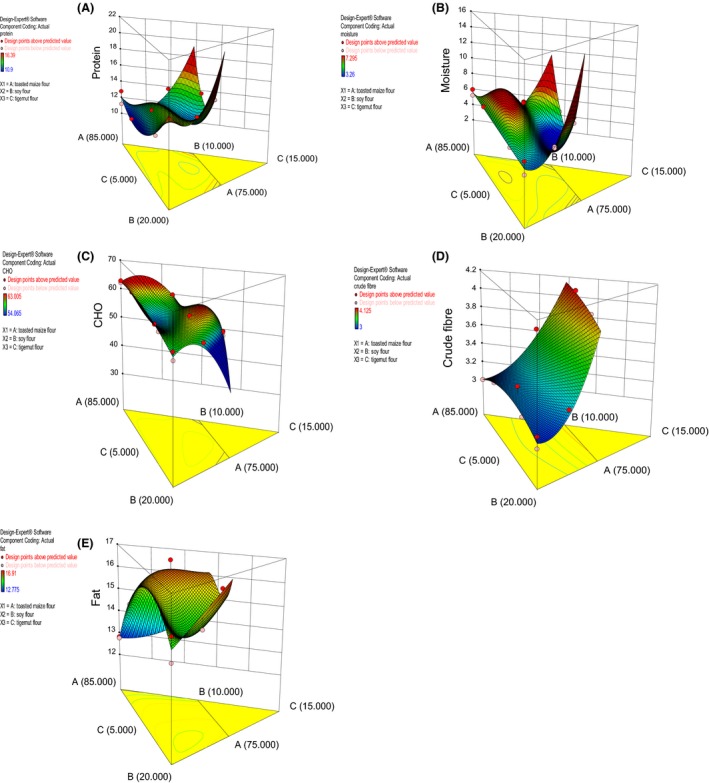
3D plot showing the effect of variables on (A) protein content, **(**B) moisture content, (C) carbohydrate (CHO), (D) crude fiber, and (E) fat.

The result of proximate composition is shown in Table 1. The protein content of the composite blends ranged from 10.9/100 g to 16.4/100 g. The highest protein percentage is recorded for sample 2 (16.4/100 g). This can be attributed to the highest ratio of soy flour percentage (20/100 g) in the flour component. Studies have also shown similar increase in protein content in soy‐composite flours (Singh et al. 2000; Mashayekh et al. 2008). It has been discovered that most leguminous plant seeds are rich in nutrients with good arrays of amino acids and minerals (Fagbemi et al. 2004). Soybean is an excellent source of protein and a complement to lysine‐limited cereal protein. The analysis of variance (ANOVA) result for protein showed the model (quadratic), and model terms (linear mixture, AB, AC, BC, A^2^BC, AB^2^C, and ABC^2^) are significant (*P *≤ 0.05). The R‐squared and Adjusted R‐squared values were 0.9503 and 0.8935, respectively, which showed that the model was good. The 3D plot showing the effect of the composite flour on protein content is shown in the Figure 1A. Soybean contributes to the protein content most. Toasted maize. probably because of its quantity, also contributes to protein content. Tigernut flour had the least contribution.

**Table 1:** Proximate composition (g/100 g) of the composite blends from toasted maize, soy, and tigernut flour

Runs	A(%)	B(%)	C(%)	Protein	Ash	Moisture	CHO	Crude fiber	Fat
1	81.702	10.000	8.298	12.9	4.6	5.0	59.9	3.5	14.1
2	75.000	20.000	5.000	16.4	4.2	3.5	58.5	3.0	14.4
3	75.117	17.308	7.576	14.9	4.2	5.2	56.4	3.3	16.0
4	77.437	17.563	5.000	12.5	4.0	4.6	59.3	3.2	16.4
5	75.117	17.308	7.576	14.8	4.3	5.0	57.4	3.3	15.2
6	78.567	11.433	10.000	14.4	4.4	5.4	55.8	4.1	15.9
7	77.290	15.138	7.572	13.3	3.8	3.3	61.5	3.3	14.8
8	85.000	10.000	5.000	11.2	3.7	6.0	63.3	3.0	12.8
9	81.702	10.000	8.298	13.8	4.7	5.2	59.6	3.6	13.1
10	75.000	20.000	5.000	16.4	4.4	5.1	55.6	3.1	15.4
11	76.299	13.701	10.000	15.0	3.7	5.7	55.4	4.0	16.2
12	85.000	10.000	5.000	12.8	3.8	5.3	62.3	3.0	12.8
13	76.299	13.701	10.000	15.1	4.6	6.4	54.0	4.0	15.9
14	80.152	12.337	7.511	12.5	3.5	6.6	57.1	3.4	16.9
15	82.540	12.460	5.000	10.9	2.6	5.5	63.0	3.1	14.9
16	79.683	14.616	5.701	13.5	3.2	7.3	56.8	3.3	15.9

A, toasted maize flour; B, soy flour; C, tigernut flour; CHO, carbohydrate.

The Ash content ranged from 2.6/100 g to 4.7/100 g. Ash is an indication of mineral contents of foods and has been discovered to be abundant in soy‐supplemented cereal meals (Alabi and Anuonye 2007). The ANOVA result of ash showed that the model (quadratic) and the model terms (linear mixture and AB) were significant (*P *≤ 0.05). The R‐squared and adjusted R‐squared values are 0.7531 and 0.6341, respectively.

The moisture content of the composite flour ranged from 3.3/100 g to 7.3/100 g. This is lower than the minimum limit of 10/100 g moisture content for flour (Ihekoronye and Ngoddy 1985), which indicated that the composite flour can be used to produce a shelf stable products (Akhtar et al. 2008; Elleuch et al. 2011). The moisture contents of flour should be less than 10% as obtained in this work. The ANOVA result for moisture content showed that the model (quadratic) and model terms (AC, BC, A^2^BC, and ABC^2^) were significant (*P *≤ 0.05). The R‐squared and adjusted R‐squared values were 0.8301 and 0.6360, respectively. The 3D plot showing the effect of the variables on the moisture is shown in Figure 1B. The figure showed that toasted maize flour was responsible for the low value of the moisture content. This could be an additional advantage of using toasted maize in composite flour development.

The carbohydrate content of the composite flour blends ranged from 54.0/100 g to 63.3/100 g. The ANOVA result for the carbohydrate content showed that the model and model terms (linear mixture, AC, BC, A^2^BC, and ABC^2^) were significant (*P *≤ 0.05) indicating that all the samples are good sources of carbohydrate. The lack of fit is also not significant. The contribution of the variables to carbohydrate is high which might result in high energy content of the composite flour (Awolu et al. 2015a) as carbohydrate content and energy content of a food product are directly proportional. The R‐squared and adjusted R‐squared values are 0.9374 and 0.8658, respectively. The 3D plot as shown in the Figure 1C also buttressed the positive effects of the variables on the carbohydrate content.

Crude fiber content ranged from 3.1/100 g to 4.1/100 g; which increased as the quantity of tigernut flour in the composite sample increases. Total dietary fiber intake for adult men and women is 38 and 25 g/day, respectively (Neha and Ramesh 2012). Soluble fibers are effective in reducing total blood cholesterol, whereas insoluble fibers controls constipation and reduce the risk of colon cancer (Islam et al. 2007). Consumption of vegetable fiber has been shown to reduce serum cholesterol level, risk of coronary heart disease, colon and breast cancer, and hypertension; enhance glucose tolerance and increase insulin sensitivity (Hassan and Umar 2004a). The ANOVA result for crude fiber showed that the model (quadratic) and model terms (linear mixture, AB, AC, and BC) were significant (*P *≤ 0.05). The lack of fit F‐value of 0.94 is not significant, whereas the R‐squared and adjusted R‐squared values were 0.9855 and 0.9783, respectively. The 3D plot showing the effect of the variables on the crude fiber as shown in Figure 1D showed that all the variables had positive effect on the fiber content.

The fat content of the composite blends ranged from 12.8/100 g to 16.9/100 g. High fat content could affect the shelf life of the composite flour due to oxidative activities (Awolu et al., 2015a, 2015b). The ANOVA result for the fat content showed that only the model (quadratic) and model terms (linear mixture and AB) were significant (*P *≤ 0.05). The R‐squared and adjusted R‐squared values are 0.9159 and 0.8198, respectively. The 3D plot showing the effect of the variables on the fat as shown in Figure 1E showed that soy flour and tigernut flour were the major contributors to fat contents.

### Mineral composition

The result of the mineral compositions of the composite blends is shown in Table 2. Potassium and calcium were the most abundant elements in the samples. Potassium protects against arterial hypertension (Wardlaw 2004). Also, potassium is required to maintain osmotic balance of the body fluids, the pH of the body, to regulate muscle and nerve irritability, control glucose absorption, and enhance normal retention of protein during growth (Omoba et al. 2013). The values of calcium found in the composite flour are adequate for bone and teeth development (Oladele and Aina 2007). Inadequate intakes of Zinc and Iron have been associated with severe malnutrition, increased disease conditions, and mental impairment (Wardlaw 2004; Mannay and Shadaksharaswany 2005). The results from mineral analysis showed that the composite flour would contribute substantially to the recommended dietary requirements for minerals.

**Table 2:** Mineral composition of composite blends of toasted maize, soy, and tigernut flour

Runs	A(%)	B(%)	C(%)	Ca (ppm)	K (ppm)	Mn (ppm)	Fe (ppm)	Cu (ppm)	Zn (ppm)
1	81.702	10.000	8.298	40.80	50.00	0.17	1.52	0.11	1.09
2	75.000	20.000	5.000	30.20	38.90	0.25	1.91	0.14	0.98
3	75.117	17.308	7.576	30.80	40.60	0.26	1.73	0.14	1.12
4	77.437	17.563	5.000	43.50	54.60	0.21	1.83	0.19	0.83
5	75.117	17.308	7.576	30.50	40.50	0.30	1.76	0.14	1.11
6	78.567	11.433	10.000	55.70	60.30	0.16	1.61	0.16	0.85
7	77.290	15.138	7.572	30.10	64.70	0.30	2.44	0.18	1.41
8	85.000	10.000	5.000	49.60	59.30	0.20	2.06	0.26	0.77
9	81.702	10.000	8.298	40.70	50.30	0.17	1.53	0.11	1.09
10	75.000	20.000	5.000	30.10	39.00	0.24	1.90	0.14	0.95
11	76.299	13.701	10.000	48.40	51.10	0.28	2.60	0.11	1.20
12	85.000	10.000	5.000	49.60	59.30	0.21	2.05	0.27	0.80
13	76.299	13.701	10.000	47.90	50.90	0.26	2.59	0.10	1.21
14	80.152	12.337	7.511	51.40	40.70	0.18	1.75	0.24	0.88
15	82.540	12.460	5.000	49.60	41.30	0.26	2.02	0.22	1.18
16	79.683	14.616	5.701	47.30	39.80	0.22	2.01	0.16	1.05

A, toasted maize flour; B, soy flour; C, tigernut flour.

Considering the results of the protein content, crude fiber content, and mineral contents, the three best blends are run 2 (75.000/100 g of toasted maize flour, 20.000/100 g of soy flour, and 5.000/100 g of tigernut flour), run 7 (77.290/100 g of toasted maize flour, 15.138/100 g of soy flour, and 7.572/100 g of tigernut flour), and run 11 (76.299/100 g of toasted maize flour, 13.701/100 g of soy flour, and 10.000/100 g of tigernut flour).

### Functional Properties

Functional properties determine the application and use of food materials for various food products. The results of functional properties of the best three blends of toasted maize, soy, and tigernut composite flour are presented in Table 3. The BD ranged from 0.77 to 0.82 g/mL. The BD is influenced by particle size and the density of the flour. It is important in determining the packaging requirement and material handling (Plaami 1997). It has been shown that high BD is desirable for greater ease of dispersibility and reduction of paste thickness (Amadinkwa 2012). It also determines the mixing quality of food materials (Lewis 1990). Low BD had been found to be an advantage in formulating complementary foods as it enhances nutrient and calorie density per feed of child (Akpata and Akubor 1999).

**Table 3:** Functional properties of toasted maize, soy, and tigernut flour composite blend

Sample	BD (g/mL)	SI	FC(%)	FS(%)	WAC (gs^−1^)	(LGC)
2	0.77	0.80	31.37	19.61	4.00	0.40
7	0.81	0.70	17.65	9.80	3.70	0.40
11	0.82	0.70	21.57	5.88	3.70	0.40

BD, bulk density; SI, solubility index; FC, foaming capacity; FS, foaming stability; WAC, water absorption capacity; LGC, least gelation concentration.

Sample 2 had the highest swelling power. Swelling index of flours depends on size of particles, type of variety, and types of processing methods or unit operation (Chandra and Samsher 2013). Also, sample 2 had the highest foam capacity. The high foam capacity of sample 2 could be as a result of its high protein content. Foaming ability has been related to the amount of solubilized protein (Narayana and Narasinga Rao 1982).

Sample 2 also had the highest water absorption capacity (4.00 g/sec). Water absorption characteristic represents the ability of a product to associate with water under conditions where water is limited. The result suggests that the composite flour samples would find application in cookies and biscuit (Oladele and Aina 2007). The LGC of the three samples was the same.

### Pasting properties

The result of pasting properties of the three best blends is presented in the Table 4. Pasting property influences quality and esthetic considerations in the food industry as they affect texture and digestibility as well as the end use of starch‐based food commodities (Adebowale et al. 2005). Sample 11 had the highest peak viscosity (222.08 RVU), whereas sample 2 had the least (199.00 RVU). The only reason that could account for the higher peak viscosity of sample 11 was the higher amount of tigernut flour in the sample blend. High peak viscosity is an indication of high starch content (Osungbaro 1990). Peak viscosity is an index of the ability of starch‐based foods to swell freely before their physical breakdown (Sanni et al. 2006; Adebowale et al. 2008).

**Table 4:** Pasting properties of toasted maize, soy, and tigernut flour composite blends values are mean of triplicate samples

Sample	Peak viscosity (RVU)	Trough	Breakdown viscosity (RVU)	Final viscosity (RVU)	Set back viscosity (RVU)	Peak time (min)	Pasting temperature (^°^C)
2	199.00	194.00	5.00	235.58	41.58	6.54	83.65
7	221.42	157.00	64.42	237.08	80.08	6.74	84.25
11	222.08	140.42	81.67	233.00	92.58	6.66	82.58

Sample 7 had highest peak time, closely followed by samples 11 and 2 in that order. Peak time is a measure of the cooking time. Pasting temperature ranged from 83.65 to 84.25°C. A high pasting temperature indicates high water‐binding capacity, high gelatinization tendency, and low swelling property of starch‐based flour due to high degree of association between starch granules (Adebowale et al. 2008). Sample 7 had highest pasting temperature followed by samples 11 and 2 in that order. Trough is the minimum viscosity value and it measures the ability of paste to withstand breakdown during cooling. The values ranged between 140.42 and 194.00 RVU with sample 2 having the highest and sample 11 lowest.

Sample 11 had the highest set back and breakdown viscosities values while sample 2 had the lowest. Sample 2 is therefore more stable to heat and mechanical shear than samples 7 and 11(Oladele and Aina 2007). Retrogradation of the flour paste decreases with increasing setback viscosity; and the lower the staling rate of the product made from the flour (Adeyemi and Idowu 1990).

The final viscosity ranged from 233.00 to 237.08 RVU with sample 7 having the highest value, followed by sample 2 and sample 11, respectively. Final viscosity is commonly used to define the quality of particular starch‐based flour, as it indicates the ability of the flour to form viscous paste after cooking and cooling. It also gives a measure of the resistance of paste to shear force during stirring (Adebowale et al. 2005, 2008).

### Antioxidant and antinutritional properties

The result of oxalate, phytate, and total phenol constituent of the three choice blends is shown in Table 5. The total oxalate ranged from 3.53 mg ± 0.02/100 g to 3.79 mg ± 0.01/100 g. Sample 2 had the lowest value of total oxalate. The values were lower than a total oxalate of 366.6 mg/100 g and a soluble oxalate of 250 mg/100 g for a multimix diet reported by Okoro (1986). Raw and processed oxalate contents of grain amaranth were 91 mg/100 g and 57 mg/100 g, respectively. Large amounts of oxalate bind with calcium‐forming calcium oxalate which is insoluble and not absorbed by the body. They are therefore considered poisonous at high concentration, but harmless when present in small amounts (Fox and Cameron 1986). High oxalate level in food has been implicated as the cause of kidney stones; it correlates with increase in calcium absorption in the kidney (Chai and Liebman 2004).

**Table 5:** Result of oxalate, phytate, and total phenol constituent of the three best blends

Sample	Oxalate (mg/100 g)	Total phenol (mg/100 g)	Phytate (mg/100 g)
2	3.53 ± 0.02	4.75 ± 0.02	5.95 ± 0.02
7	3.79 ± 0.01	4.63 ± 0.01	6.07 ± 0.01
11	3.54 ± 0.02	4.56 ± 0.02	5.78 ± 0.01

Phytates ranged from 5.78 mg ± 0.01/100 g to 6.07 mg ± 0.01/100 g. These antinutrients chelate divalent cations, such as calcium, magnesium, and iron, reducing their bioavailability (Sandberg 2002). Toxicity of oxalates and phytates for humans was set at 2–5 g/day and the consumption of diet rich in these antinutrients may result in kidney disease (Hassan and Umar 2004b; Hassan et al. 2007). The oxalate and phytate levels of the flour in this work are safe for human consumption.

Total phenolic content in sample 2 (4.75 ± 0.02 mg/100 g) was the highest. This was followed by sample 7 (4.63 ± 0.01 mg/100 g) and then sample 11 (4.56 ± 0.02 mg/100 g). This result is similar to the values obtained for Marama‐sorghum composite flours and porridges by Kayitesi et al. (2012). The high phenolic content could be attributed to the toasting of the maize grains before flour processing. Kayitesi et al. (2012) reported that dry heating Marama beans prior to its processing increased total phenolic content and antioxidant activity of the flours. Increase in total phenolic content and antioxidant activity following roasting peanuts was also reported by Talcott et al. (2005). Dewanto et al. (2002) reported that thermal processing may release bound phenolics from cellular constituents. In addition, heating induces production of Maillard reaction products, melanoidins, which have been reported to have antioxidant activity (Michalska et al. 2008). High phenolic content leads to high antioxidant activity. Free radicals and other reactive oxygen species contribute to the development of many diseases (Shahidi and Naczk 2004). Phenolic compounds scavenge free radicals by donating hydrogen atoms to free radicals; hence, may protect cell constituents against oxidative damage and limit the risk of various degenerative diseases associated with oxidative stress (Anderson and Wolf 1995).

### Amino acid profile

The amino acid profile of the three best blends is shown in Table 6. Essential and non‐essential amino acid contents of the flour were determined. Sample 2 was the highest in all the amino acids. This could be as a result of soy protein influence on the composite. Sample 2 had the highest soy composition. Glutamic acid and valine were the most abundant amino acids in the three flour blends.

**Table 6:** Amino acid profile of the three best blends

Amino acid	Sample 2 (mg/100 g)	Sample 7 (mg/100 g)	Sample 11 (mg/100 g)
Valine	1889.22	1777.55	1669.25
Threonine	377.74	350.25	336.36
Isoleucine	557.35	525.28	562.25
Leucine	1126.25	1077.25	1114.25
Methionine	163.36	157.57	150.25
Phenylalanine	785.85	740.12	705.25
Lysine	1332.25	1263.36	1244.25
Histidine	444.25	420.25	433.36
Tryptophan	238.38	220.02	221.15
**Essential AA**	**6914.65**	**6531.65**	**6436.37**
Alanine	654.54	625.25	640.36
Aspartate	196.36	192.25	189.65
Glutamate	2244.25	2228.33	2202.25
Serine	1166.33	1144.22	1098.85
Glycine	667.67	650.42	625.58
Proline	666.33	642.25	635.35
Arginine	685.85	657.57	633.36
Tyrosine	374.22	340.45	355.58
Cysteine	119.63	112.25	109.65
**Nonessential AA**	**6775.18**	**6592.99**	**5850.27**
**Total AA**	**3689.83**	**13124.64**	**12286.64**

AA, Amino acid.

Lysine is a major limiting amino acid in maize. Lysine content in sample 2 (1332.24 ± 0.01 mg/100 g) was significantly higher than samples 7 and 11. The main role of lysine is to participate in protein synthesis, thus it is important for growth and maintenance of the body. All essential amino acids are considerably present in the three samples. Amino acids are important components for healing and protein synthesis; any deficiency in these essential amino acids will hinder recovery process (Zuraini et al. 2006). According to Witte et al. (2002), glycine together with other essential amino acids such as alanine, arginine, and phenylalanine forms a polypeptide that will promote growth and tissue healing.

### Sensory evaluation of cookies

The sensory evaluation of cookies prepared from maize, soy, and tigernut flour presented in Table 7 showed that sample 2 was rated highest in terms of overall acceptability. The results also showed that increase in the level of tigernut flour in the composite improved crispiness and texture of the cookies. The incorporation of full fat soybean flour resulted in better taste and flavor. Sample 11 had the lowest values of 6.50 and 6.00 for taste and flavor, respectively; whereas sample 2 (20% soy flour) had the highest values of 7.90 and 7.00 for taste and flavor, respectively. There are no significant (p≤0.05) in appearance, color, taste, texture and crispiness. Significant differences (p≤0.05) however exist in flavour and overall acceptability.

**Table 7:** Result of sensory evaluation of cookies produced from the three best blends

Sample	Appearance	Color	Taste	Texture	Flavor	Crispiness	Overall acceptability
2	7.10^a^	6.30^a^	7.90^a^	6.40^a^	7.00^b^	7.30^a^	8.00^a^
7	7.10^a^	6.00^a^	6.60^a^	6.80^a^	6.70^a^	7.40^a^	7.70^ab^
11	6.50^a^	5.60^a^	6.50^a^	7.50^a^	6.00^ab^	7.60^a^	7.30^b^

Consumer quality is a major factor for selecting a product and among the main characteristics related to quality are texture, taste, and surface color of a biscuit (Omoba et al. 2013).

## Conclusion

Composite flour from toasted maize, soybean, and tigernut could serve as a good substitute to a complete wheat flour in cookies production. The sensory evaluation was rated high which is an indication of high acceptability. In particular, composite flour with 75% toasted maize, 20% soybean, and 5% tigernut (sample 2) showed exceptional flour and nutritional quality. This blend had the overall best proximate composition, essential amino acid profile, antioxidant (total phenol), functional properties, and the least antinutritional content (precisely, oxalate content). However, blend with 76.3% toasted maize, 13.7% soybean, and 10% tigernut (sample 7) was next to sample 2 in terms of the properties mentioned. In addition, sample 7 had the best mineral composition and pasting properties. The samples could be used in the production of nutritionally rich cookies with strong antioxidant property which may be better alternatives to 100% wheat flour cookies.

## Conflict of Interest

Authors declare that there is no conflict of interest.
